# Synthesis, Characterization and Antitumour Activity of Some Butyltin(IV) Cysteaminates and N,N-Dimethylcysteaminates

**DOI:** 10.1155/MBD.2000.233

**Published:** 2000

**Authors:** Marcel Gielen, Karel Handlir, Martin Hollein, Dick de Vos

**Affiliations:** 1 Departement of General and Organic Chemistry AOSC Faculty of Applied Sciences Free University of Brussels VUV Pleinlaan 2 Brussels B-1050 Belgium; 2 Departement of General and Inorganic Chemistry Faculty of Chemical Technology University of Pardubice Nam. Cs. legii 565 Pardubice 53210 Czech Republic; 3 Pharmachemie B. V. Haarlem The Netherlands

## Abstract

The synthesis and characterization of four di- and tri-n-butyltin cysteaminates and N,N-dimethylcysteaminates and three protonated / quaternized derivatives are reported. They all exhibit moderate or high *in vitro* cytotoxic activity. Six of seven compounds presented in this work are more active than cisplatin, etoposide and 5-fluorouracil, but less active than methotrexate and doxorubicin.


### SYNTHESIS, CHARACTERIZATION AND ANTITUMOUR ACTIVITY OF SOME BUTYLTIN(IV) CYSTEAMINATES AND N,N-DIMETHYLCYSTEAMINATES

Marcel Gielen[1], Karel Handlir*[2], Martin Hollein[2], and Dick de Vos[3]

[1] Departement of General and Organic Chemistry AOSC, Faculty of Applied Sciences, Free University of Brussels VUV, Pleinlaan 2, B-1050 Brussels, Belgium
[2] Departement of General and Inorganic Chemistry, Faculty of Chemical Technology, University of Pardubice, Nam. Cs. legii 565, 53210 Pardubice, Czech Republic
[3] Pharmachemie B. V., Haarlem, the Netherlands

**Abstract.**
The synthesis and characterization of four di- and tri-n-butyltin cysteaminates and N,N-dimethylcysteaminates and three protonated / quaternized derivatives are reported. They all exhibit moderate or high *in vitro* cytotoxic activity. Six of seven compounds presented in this work are more active than cisplatin, etoposide and 5-fluorouracil, but less active than methotrexate and doxorubicin.

### Introduction.

Several organotin cysteaminates (aminoethylthiolates) have been studied[1-4] as compounds with potentially high biological activity, but little is known about their cytotoxic effect. The present paper reports the synthesis and characterization of di- and tri-n-butyltin cysteaminates and their N,N-dimethyl analogues and their *in vitro* cytotoxicity.
It is shown that a correct evaluation of the biological activity of organotin compounds remains often hampered by their low water solubility[5]. Therefore, we focused on the synthesis of derivatives with potentially higher water solubility by introducing ionic groups into the molecule by protonation or quaternization of a nitrogen atom.

### Results and discussion.

3 1 2 4

$(n\text{-}Bu)_n\ Sn\ [SCH_2\text{-}CH_2\text{-}NH_m(CH_3)_{2-m}]_{4-n}$

| | | |
|---|---|---|
| $n = 3$, | $m = 2$ | **(1)** |
| $n = 3$, | $m = 0$ | **(2)** |
| $n = 2$, | $m = 2$ | **(3)** |
| $n = 2$, | $m = 0$ | **(4)** |

3 1 2 4

$(n\text{-}Bu)_3\ Sn\ S\text{-}CH_2\text{-}CH_2\text{-}NH_m(CH_3)_{3-m}\ X$

| | | |
|---|---|---|
| $m = 3$, | $X = CH_3SO_3$ | **(5)** |
| $m = 1$, | $X = CH_3SO_3$ | **(6)** |
| $m = 0$, | $X = I$ | (7) |

δ γ β α

$n\text{-}Bu = CH_3\text{-}CH_2\text{-}CH_2\text{-}CH_2$

Tri-n-butyltin cysteaminate (**1**), tri-n-butyltin N,N-dimethylcysteaminate (**2**), di-n-butyltin bis(cysteaminate) (**3**) and di-n-butyltin bis(N,N-dimethylcysteaminate) (**4**) were prepared by the reaction of the sodium cysteaminate (prepared *in situ* by the reaction of sodium methylate with the hydrochloride of cysteamine) with the suitable di- or tri-n-butyltin chloride in chloroform following reference [1].
The methanesulphonates **5** and **6** were respectively synthesized by the reactions of the unprotonated analogs **1** and **2** with an equimolar amount of methane sulphonic acid in chloroform. After purification on a short column of alumina and evaporation of the solvent, colourless viscous oils were obtained. The quaternized methyl derivative of **2**, the methiodide (**7**), was prepared by its reaction with methyl iodide in benzene and purified by recrystalization from chloroform.

Table 1. Melting points and yields for compounds **1** – **7**.

| comp. | formula[1,2] | m.p., b.p.,°C | yield,% |
|---|---|---|---|
| **1** | $(C_4H_9)_3SnSCH_2CH_2NH_2$ | 120-123/40 Pa | 86 |
| **2** | $(C_4H_9)_3SnSCH_2CH_2N(CH_3)_2$ | 129-131/35 Pa | 77 |
| **3** | $(C_4H_9)_2Sn(SCH_2CH_2NH_2)_2$ | 167-169/35 Pa | 85 |
| **4** | $(C_4H_9)_2Sn(SCH_2CH_2N(CH_3)_2)_2$ | 172-174/40 Pa | 90 |
| **5** | $(C_4H_9)_3SnSCH_2CH_2NH_3\ CH_3SO_3$ | oil | 92 |
| **6** | $(C_4H_9)_3SnSCH_2CH_2NH(CH_3)_2\ CH_3SO_3$ | oil | 94 |
| **7** | $(C_4H_9)_3SnSCH_2CH_2N(CH_3)_3\ I$ | 118-120 | 74 |

[1] The elemental analyses (C, H, N, Sn) agree satisfactorily with the proposed formulas.

Compounds **1** – **4**, and **7**, are poorly soluble in aqueous solutions (< 1mg/mL). Compounds **5** and **6** exhibit higher water solubilities (ca. 2 mg/mL). Compounds **1** - **7** are soluble in a therapeutic solution (0,103 M solution of NaCl in water/DMSO 1/9). A white turbidity appears in these solutions only after several hours due to slow hydrolysis.

Compounds **1** - **7** were characterized by multinuclear NMR spectroscopy. The NMR parameters are given in Table 2.

Table 2. Resonances of $^{13}C$, $^{15}N$ and $^{119}Sn$ NMR spectra of the compounds **1-7** in $CDCl_3$ (ca 20% sol. v/v).

| compound | **1** | **2** | **3** | **4** | **5** | **6** | **7** |
|---|---|---|---|---|---|---|---|
| $\delta(^{119}Sn)$, ppm | 77.6 | 77.5 | -28.9 | 38.6 | 86.3 | 89.5 | 93.2 |
| $\delta(^{13}C)^a$, ppm α | 12.3 | 12.75 | 22.38 | 18.78 | 13.45 | 13.45 | 13.60 |
| | (332) | (331) | (495) | (438) | (322) | (350) | (339) |
| β | 27.93 | 27.97 | 28.27 | 27.51 | 28.30 | 28.34 | 28.47 |
| | (21.3) | (21.1) | (28.4) | (26.4) | (21.4) | (21.3) | (22.0) |
| γ | 26.32 | 26.38 | 26.42 | 25.90 | 26.85 | 26.81 | 26.91 |
| | (59.6) | (59.8) | (87.1) | (83.8) | (62.7) | (61.4) | 62.2) |
| δ | 12.89 | 12.95 | 13.35 | 12.82 | 13.45 | 13.58 | 14.00 |
| | (-)[c] | (-)[c] | (-)[c] | (-)[c] | (-)[c] | (-)[c] | (-)[c] |
| 1 | 30.31 | 23.59 | 30.04 | 23.84 | 23.92 | 20.94 | 19.70 |
| 2 | 45.02 | 63.12 | 44.44 | 61.84 | 43.08 | 60.94 | 69.49 |
| 3 | | 44.71 | | 44.46 | | 43.42 | 54.18 |
| 4 | | | | | 39.15 | 39.16 | |
| $\delta(^{15}N)$, ppm | -360.4 | -354.0 | -355.3 | -354.4 | -349.4 | -344.2 | -325.8 |
| $\Delta\delta$ b | 1.1 | 1.5 | 6.2 | 1.5 | 12.7 | 11.7 | 30.1 |

[a] values of $^nJ(^nJ\ ^{119}Sn, ^{13}C)$ coupling constants in Hz for carbon atoms in parentheses

[b] difference between $\delta(^{15}N)$ for the organotin compound and free cysteamine, $\delta(^{15}N)$ = 361.5 ppm; N,N-dimethylcysteamine, $\delta(^{15}N)$ = -355.9 ppm)

[c] not observed

$^{13}C$ resonances were assigned on the basis of the values of $^nJ(^{119}Sn,^{13}C)$ coupling constants and standard $^{13}C$ - APT techniques utilization in agreement with ref.[1].

The values of $^{119}Sn$ chemical shifts are found in the interval characteristic for four-coordinated tin[6,7]. The values of the coupling constants $^1J(^{119}Sn,^{13}C)$ agree with the structure proposed. Compound **3** is characterized by a somewhat larger upfield shift, close to the upper limit of the above mentioned interval, together with a high value of $^1J(^{119}Sn,^{13}C)$ (495 Hz). The C–Sn-C angle, estimated[6,7] from this value of coupling constant is 124°. According to ref.[1], this behaviour can be due to intermolecular association increasing the coordination number of tin in concentrated solutions, as suggested by the concentration dependence of $\delta(^{119}Sn)$ and cryoscopic measurements in benzene.

The values of the $\delta(^{15}N)$ chemical shifts of compounds **1**, **2** and **4** only slightly differ from the $\delta(^{15}N)$ values of the free cysteamine. The significantly larger $\delta(^{15}N)$ chemical shift of compound **3** is however much lower than those of compounds **5**, **6** and **7** containing a tetracoordinated nitrogen atom. It can be stated that the Sn → N interaction in compounds **1** – **4** is negligible or only very weak in chlorofom solutions. This conclusion is not in contradiction with the proved Sn → N interaction found in the solid state[8].

**Antitumour activity.**

The results of the *in vitro* antitumour tests of compounds **1** – **7** are given in Table 3 as the inhibition doses $ID_{50}$ observed against a panel of seven human tumour cell lines, MCF-7 and EVSA-T, two breast cancers, WiDr, a colon cancer, IGROV, an ovarian cancer, M19 MEL, a melanoma, A248, a renal cancer, and H226, a non-small cell lung cancer. The antitumour tests results are compared with those obtained for clinically used reference compounds[9] like cisplatin, doxorubicin, etoposide, 5-fluorouracil and methotrexate.

Table 3. Inhibition doses $ID_{50}$ of compounds **1** – **7** and of five reference compounds.

| comp. | MCF-7 | EVSA-T | WiDr | IGROV | M19MEL | A498 | H226 |
|---|---|---|---|---|---|---|---|
| **1** | 41 | 33 | 39 | 44 | 67 | 85 | 39 |
| **2** | 41 | 35 | 39 | 46 | 78 | 88 | 39 |
| **3** | 51 | 45 | 211 | 68 | 72 | 118 | 70 |
| **4** | 78 | 65 | 331 | 110 | 112 | 129 | 108 |
| **5** | 30 | <3 | 19 | 29 | 31 | 42 | 36 |
| **6** | 90 | 57 | 40 | 83 | 77 | 133 | 111 |
| **7** | 385 | 367 | 339 | 411 | 327 | 758 | 576 |
| cisplatin | 699 | 422 | 967 | 169 | 558 | 2 253 | 3 369 |
| doxorubicin | 10 | 8 | 11 | 60 | 16 | 90 | 199 |
| etoposide | 2 594 | 317 | 150 | 580 | 505 | 1 314 | 3 934 |
| 5-fluorouracil | 750 | 475 | 225 | 297 | 442 | 143 | 340 |
| methotrexate | 18 | 5 | <3 | 7 | 23 | 37 | 2 287 |

It is evident from Table 3 that all studied compounds exhibit excellent cytostatic activities, except compound **7** showing only moderate activity. Compounds **1** - **6** are more active than cisplatin and, in most cases, than etoposide and 5-fluorouracil. Their activity is lower than that of doxorubicin and methotrexate in most cases for the studied cancer cell lines. The very limited set of acquired activity values show that the activity of tri-n-butyltin compounds are higher than those of di-n-butyl analogues (**1**>**3**, **2**>**4**) for all cell lines, especially against WiDr. The N,N-dimethyl derivatives exhibit an activity comparable to or somewhat higher than that of unmethylated compounds. Compound **8**, with a quaternized nitrogen, exhibits the lowest cytotoxic activities, approximatively 9 times lower than compound **2**. It might be underlined that the ionic more water-soluble compounds **5** and **6** are not more active than the uncharged analogs **1-4**, whereas the hydrophilic organotin polyoxacarboxylates exhibit much higher *in vitro* activities than other organotin carxylates[5,11]. Compounds **5** and **6** (with a protonated nitrogen) exhibit activities comparable or even considerably higher (compound **5**) against EVSA-T than the corresponding unprotonated compounds. Compound **7** (with a quaternized nitrogen) exhibits the lowest cytostatic activities, approximately 9 times lower than compound **2**. It should however be mentioned that another ionic organotin compound, bis(dicyclohexyl)ammonium bis(2,6-pyridinecarboxylato)di-n-butylstannate, where the organotin moiety is this time anionic, is also as active as the uncharged analog[12].

**Instrumentation.**

All NMR spectra were recorded on a Bruker AMX 360 instrument using a 5 mm multinuclear tuneable probe. The residual $CHCl_3$ resonance at 7.24 ppm was used as reference for the $^1H$ spectra and the central $^{13}CDCl_3$ resonance at 77.0 ppm, for the $^{13}C$ spectra. The $^{119}Sn$ chemical shifts were refered to the external tetramethyltin [$\delta(^{119}Sn) = 0.0$]. The $^{15}N$ NMR spectra were measured using the INEPT technique or in inverse-gated mode. (reference: external nitromethane, [$\delta(^{15}N) = 0.0$]).

The protocol followed for the *in vitro* antitumour screenings has been already reported[10].

**Acknowledgements.**

K. H. and M.H. thank the Grant Agency of the Czech Republic (Grant No. 203/00/0920) and the Ministry of Education, Youth and Sport of the Czech republic, associated with EU in the COST 8.20 program for financial support of this work. We are grateful to Mr. R. G. Oostrum, Dr. J. Verweij, Prof. Dr. G. Stoter, r. K. Nooter, Laboratory of Experimental Chemotherapy and Pharmacology, Department of Medical Oncology, Rotterdam Cancer

Institute, NL - 3008 AE, Rotterdam, The Netherlands, for performing the *in vitro* tests. This research was supported by the Fund for Scientific Research Flanders (Belgium), grant nr G.0074.00, M. G.).

**References.**

1. B. S. Saraswat, J. Mason, *Polyhedron(9),* **5** (1986), 1449.
2. G. Domazetis, R. J. Magee, B. D. James, *J. Organomet. Chem,* **162** (1978), 239.
3. J. D. Cashion, G. Domazetis, B. D. James, *J. Organomet. Chem,* **185** (1980), 433.
4. G. Domazetis, R. J. Magee, B. D. James, *J. Organomet. Chem,* **148** (1978), 339.
5. G. Atassi, *Rev. Si Ge Sn Pb Cpds,* **8** (1985), 219; M. Kemmer, M. Gielen, M. Biesemans, D. de Vos, R. Willem, *Metal-Based Drugs* 5 (1998), 189; M. Gielen, M. Biesemans, D. de Vos, R. Willem, *J. Inorg. Biochem.*, **79** (2000), 139.
6. J. Holecek, A. Lycka, *Inorg. Chim. Acta,* **118** (1986), L15.
7. J. Holecek, M. Nadvornik, K. Handlir, A. Lycka, *J. Organomet. Chem.,* **315** (1986), 299.
8. B. D. James, R. J. Magee, W. C. Patalinghug, B. W. Skelton, A. H. White, *J. Organomet. Chem,* **467** (1994), 51
9. P. Skehan, R. Storeng, D. Scudiero, A. Monks, J. McMahon, D. Vistica, J. T. Warren, H. Bokesh, S. Kenney, M. R. Boyd, *J. Natl. Cancer Inst.,* **82** (1990), 1107.
10. Y. P. Kepers, G. J. Peters, J. Van Ark-Otte, B. Winograd, H. M. Pinedo, *Eur. J. Cancer,* **27** (1991), 897.
11. M. Kemmer, L. Ghys, M. Gielen, M. Biesemans, E. R. T. Tiekink, R. Willem, *J. Organomet. Chem.* **582** (1999), 195.
12. S. W. Ng, V. G. Kumar Das, J. Holecek, A. Lycka, M. Gielen, M. G. B. Drew, *Appl. Organomet. Chem.* **11** (1997), 39.

**Received: September 22, 2000 - Accepted: October 19, 2000 - Received in revised camera-ready format: October 20, 2000**

